# Biodegradation Capabilities of Paraquat-Degrading Bacteria Immobilized on Nanoceramics

**DOI:** 10.3390/toxics11070638

**Published:** 2023-07-23

**Authors:** Manee Jindakaraked, Eakalak Khan, Puangrat Kajitvichyanukul

**Affiliations:** 1 Department of Environmental Engineering, Faculty of Engineering, Chiang Mai University, Chiang Mai 52000, Thailand; rammy55063390@gmail.com; 2 Civil and Environmental Engineering and Construction Department, University of Nevada, Las Vegas, NV 89154-4015, USA; eakalak.khan@unlv.edu

**Keywords:** nanoceramic, nanoclay, biodegradation, cell immobilization, pesticide, mineralization

## Abstract

The biodegradation of paraquat was investigated using immobilized microbial cells on nanoceramics fabricated from nanoscale kaolinite. *Pseudomonas putida* and *Bacillus subtilis*, which degrade paraquat, were immobilized separately on nanoceramics (respectively called IC_nc_−P and IC_nc_−B). The attachment of bacteria to nanoceramics resulted from electrostatic force interactions, hydrogen bonding, and covalent bonding (between the cells and the support materials). The initial 10 mg L^−1^ concentration of paraquat in water was removed by the adsorption process using nanoceramics at 68% and ceramics at 52%, respectively. The immobilized cells on the nanoceramics were able to remove approximately 92% of the paraquat within 10 h, whereas the free cells could only remove 4%. When the paraquat was removed, the cell−immobilized nanoceramics exhibited a significant decrease in dissolved organic nitrogen (DON). IC_nc_−B was responsible for 34% of DON biodegradation, while IC_nc_−P was responsible for 22%. Ammonia was identified as the end product of ammonification resulting from paraquat mineralization.

## 1. Introduction

Paraquat, widely used as a herbicide, is well−known as an extremely toxic chemical with a human median lethal dose (LD_50_) of 3–5 mg kg^−1^ [1]. Environmental contamination from paraquat, especially in Thailand’s soil and water has been reported [2]. Strong soil binding makes paraquat comparatively immobile. Approximately 0.1% of the applied paraquat will be present in the soil water. The half−life varies from 1.4 to 7.2 years, depending on the soil composition [3]. Paraquat can inhibit culturable soil bacteria, reduces hydrogenase activity, increases urease activity, and has a negative effect on soil fungi [4,5,6]. It can enter the food chain when animals forage for food [7]. Long−term exposure to paraquat will result in detrimental biomagnification for both humans and animals [8]. Paraquat can enter the body via the respiratory system, digestive tract, and mucosal absorption, causing varying degrees of toxicity [9].

Paraquat has been removed from the environment using a variety of techniques. Physico−chemical processes, particularly adsorption and advanced oxidation processes, are highly effective at removing toxic chemicals [10,11]. However, these techniques necessitate a relatively high initial investment and material expense [10]. Bioremediation has evolved into an attractive and effective method for removing toxic waste from polluted environments. It is highly involved in the degradation, elimination, immobilization, or detoxification of various chemical wastes and physical hazardous materials from the environment via the all−encompassing action of microorganisms. Therefore, bioremediation is the most efficient, cost−effective, and environmentally friendly method for managing a polluted environment [12]. Cell immobilization as an advanced bioremediation technique has been introduced for degrading toxic chemicals, including paraquat [13]. Specifically, the immobilization of microbial cells on adsorbent materials is well recognized as a combination technology between adsorption and biodegradation [14].

To remove paraquat from polluted water, clay minerals such as montmorillonite, nontronite, and kaolinite are among potent adsorbents [15,16,17,18,19]. Recently, many researchers have gained attention from clay−based nanomaterials with specific chemical and biological properties. Nanoclay is a very small dimension of a specific clay and has proven to be an excellent adsorbent in removing heavy metals and organics [20,21,22,23,24]. Due to the minute size and large amount of surface area per unit volume, and surface modification potential, nanoclay is more efficient as a sorbent for water contaminants than traditional clay [24]. Montmorillonite and kaolinite are the clays most used as nano−adsorbents. Taha and Mobasser (2015) [25] reported that montmorillonite was an exceptional adsorbent in dichlorodiphenyltrichloroethane (DDT) and polychlorinated biphenyl (PCB) removal from contaminated soil and solution. Ten percent of nanoclay (Cloisite^®^ Na^+^, which is a natural montmorillonite) adsorbed 75% of PCB after 16 h of equilibrium time [25]. Rezvani and Taghizadeh (2018) [24] studied the adsorption of nitrate, lead, arsenic, and turbidity from water using nanoclay granules. Comparatively, the investigated nanoclay materials (50% of nanoclay and 50% clay by mass) have higher ion exchange capacity and contaminant removal than the corresponding clay materials (100% clay). Yue et al. (2022) [26] also reported an environmentally friendly nanoclay/sodium algenate washing agent that can effectively decontaminate oiled sand.

To acquire a high efficiency in pollutant degradation and to maximize contaminant bioavailability in cell immobilization, many materials, such as polyvinyl alcohol [27], PVA–alginate–kaolin gel beads [15], alginate [28], and biochar [29,30], have been used to support the microorganisms for biodegradation purpose. A novel environmentally friendly risk−based remediation technology has emerged that uses clay minerals in integrating microorganisms [14]. The use of clay or nanoclay in conjunction with cell immobilization (i.e., PVA–alginate–kaolin gel beads) to enhance the removal of crystal violet (CV) in water was studied [15]. Entrapping *Burkholderia vietnamiensis* C09V in the PVA–alginate–kaolin gel beads provided 98% removal of CV, while the CV removal by the naked beads and free cells was only 77% and 94%, respectively [15].

Immobilization of appropriate microorganisms could enhance paraquat degradation efficiency [14]. The high biomass and strong resistance to toxic chemicals of the consortium of immobilized cells contribute to the high metabolic activity of pollutant degradation [31]. According to Huang et al. (2019) [32], some bacterial and fungal species can degrade paraquat in soils and slurry. These paraquat−degrading microbial strains are *Pseudomonas putida* [33]; *Agrobacterium tumefaciens*, *Aerobacter aerogenes*, *Pseudomonas fluorescens*, and *Bacillus cereus* [34]; *Enterobacter cloacae* PQ02 [35]; and *Aeromonas veronii* NK67 [36]. They can effectively degrade paraquat and utilize it as a carbon and/or nitrogen source to grow [34,37]. The biodegradation rate under controlled conditions is affected by multiple variables, including temperature, pH, nutrients, initial concentration, inoculum size, and bacterial or fungal strain characteristics [32].

To date, no study has reported on applying nanoclay in supporting microorganisms on pesticide degradation, especially paraquat. From past works, the immobilization of paraquat−degrading bacteria on nanoclay should enhance the paraquat removal from contaminated water. This work demonstrates for the first time the paraquat removal by nanoclay adsorption and the paraquat degradation by the immobilization of paraquat−degrading bacteria onto the nanoclay. 

In this work, the nanoclays were used innovatively to make ceramic rings (called nanoceramics), and the paraquat−degrading microorganisms, *Pseudomonas putida* TISTR 1522 (or *P. putida*) and *Bacillus subtilis* TISTR 1248 (or *B. subtilis*), were individually immobilized onto the nanoceramics (called bio−nanoceramics) for paraquat removal from a synthetic medium. The nanoceramics played a role as an excellent adsorbent as well as a cell support material. As far as we know, this is the first research study to report nanoceramics’ potential as immobilization supports for bacterial cells with a capability in paraquat adsorption and biodegradation, simultaneously.

This work focused on paraquat degradation and mineralization by bio−nanoceramics (cell−immobilized nanoceramics). The removal efficiency as well as the degradation kinetics of paraquat in a synthetic medium was examined. In addition, the reduction of dissolved organic nitrogen (DON) was used as a biomineralization efficiency indicator of paraquat.

## 2. Materials and Methods

### 2.1. Chemicals, Materials, and Culture Media

Kaolinite, polystyrene foam, and clay minerals were obtained from a domestic market, Thailand. Commercial grade paraquat was purchased from Ag−gro (Thailand) Co., Ltd., (Chiang Mai, Thailand). The active ingredients were 1,1′−dimethyl−4,4′−bipyridinium dichloride at 27.6 percent weight−per−volume, water at 30 to 60 percent, emulsifiers at 10 to 29 percent, and other non−hazardous ingredients at less than 1 percent. The emetic and dye without percentage contribution were also listed as ingredients in the paraquat chemicals. Potassium phosphate dibasic (K_2_HPO_4_) and magnesium sulfate (MgSO_4_∙7H_2_O) were analytical reagent (AR) grade and acquired from Loba Chemie Pvt. Ltd., India. Sodium chloride (NaCl), ammonium fluoride (NH_4_F), and ammonium phosphate (NH_4_PO_4_) were AR grade and purchased from EMSURE^®^, Germany. Halloysite nanoclay, 25% glutaraldehyde (C_5_H_8_O_2_), 4% osmium tetroxide (OsO_4_), ethanol, hexane, and hexamethyldisilazane (HMDS) were obtained from Sigma−Aldrich, USA. The Thailand Institute of Scientific and Technological Research Culture Collection provided *P. putida* strain TISTR 1522 and *B. subtilis* strain TISTR 1248, which are the paraquat−degrading microorganisms [13,33]. Plate Count Agar (PCA) and Luria−Bertani broth (LB broth) were purchased from HiMedia laboratory Pvt. Ltd., India.

### 2.2. Nanoceramic Synthesis

Nanoclay (kaolinite), biochar, and polystyrene foam were mixed at a weight ratio of clay:biochar:foam of 6:0.5:0.5. The mixture (200 g total) was added to 70 mL of tap water, mixed, then incubated at 28 °C overnight. Subsequently, the incubated mixture was slip cast into a ring−shaped ceramic, 1.9 ± 0.15 cm in diameter and 2.5 ± 0.22 cm in length, before drying under natural sunlight for 2–3 d. The ceramic rings were calcined at 800 °C for 2 h and sterilized by autoclave at 121 °C for 15 min before use. 

For ceramic surface modification, 32 g of the sterile ceramics were dipped into 200 mL of 1M NH_4_F solution for 30 min, then washed with sterilized deionized (DI) water 3–4 times. After that, they were dehydrated at 80 °C for 4 h in an oven and then stored in a sterile box for further use. The obtained ceramics were called nanoceramics (labeled as C_nc_). Furthermore, the regular kaolinite (non−nano size) following the same procedure as C_nc_ and also calcinated at 800 °C and labeled as C_c_ were used to produce the control ceramics.

### 2.3. Cell Immobilization on Ceramic Materials

The acclimatized cells with paraquat were used to prepare a cell suspension. The freeze−dried bacterial cells (*B. subtilis* or *P. putida*) were activated in a synthetic medium (0.057 g NH_4_PO_4_, 0.017 g K_2_HPO_4_, 0.043 g NaCl, and 0.043 g MgSO_4_.7H_2_O in 1 L of sterile distilled water), mixed with 10% LB broth and 10 mg L^−1^ paraquat, and incubated−shaken at 120 rpm, 28 °C, for 6 d. This cell solution was sub−cultured on a PCA plate that had been mixed with 10 mg L^−1^ paraquat and then incubated at 37 °C for 24–48 h. Prior to immobilization, 5 active colonies of the acclimated cells from the PCA were incubated in 500 mL of fresh LB broth on a shaker (120 rpm) at 28 °C for 18 h (called cell suspension). The initial cell adhesion was measured by the plate count technique. Briefly, 1 mL of the cell suspension was diluted in 9 mL sterile phosphate−buffered saline (PBS) and well mixed (labeled as 10^−1^). A ten−fold serial dilution from 10^−1^ to 10^−7^ was conducted. After that, 0.1 mL of the sample was put on a PCA plate. Dilutions were duplicated and three dilutions at 10^−5^ to 10^−7^ were further used. All the sample plates were incubated for 24 h at 37 °C. The total number of colonies was counted, calculated, and reported (CFU mL^−1^) as in Equation (1) [38].

For cell immobilization, a ratio of C_nc_ (g) and cell suspension (mL) of 1:10 was applied. Briefly, the sterile ceramics were dropped into the cell suspension (approximately 10^7^ CFU mL^−1^ of the initial cell concentration) and incubated−shaken (100 rpm) at 28 °C for 2 h. The cell−immobilized ceramics, called bio−nanoceramics (labeled as IC_nc_), were dried for 10 min at room temperature. Then, the first adhered cells on IC_nc_ were enumerated using the plate count technique and reported (CFU g^−1^) as in Equation (2). Briefly, one loaf of sample (3.2 g) was mashed to powder. The powder sample was added to 9 mL sterile PBS (labeled as 10^−1^). Then, a 10−fold serial dilution from 10^−1^ to 10^−6^ was performed and spread on PCA plates in the same manner as the cell suspension as described above [38].

(1) $$CFU/mL=\frac{average\;colony}{0.1mL}dilution\;factor$$

(2) $$CFU/g=\frac{\frac{average\;colony}{0.1mL}}{g\;of\;bio-nanoceramic}dilution\;factor$$

### 2.4. Characterization of Nanoceramic and Bio−Nanoceramic

A scanning electron microscope (SEM) (Leo1455VP, Leo Electronics Co., Ltd., Tokyo, Japan) was used to examine the morphology of the cells and ceramic surfaces (of IC_nc_). Sample preparation followed a slightly modified method from [39]. The sample size was 1.5 to 2 mm in diameter and 0.4 to 0.6 mm in height. Three steps of the sample preparation process are as follows. In the fixation step, 2.5% glutaraldehyde was dropped on the sample for 1 h, followed with 1% osmium tetroxide for 2 h. In the dehydration step, 30% ethanol was first dropped on the samples for 15 min prior, followed by a concentration of 50%, 70%, 90%, and 100%, respectively. Finally, for the drying step, the sample was soaked in hexamethyldisilazane for 5 h. All the steps were conducted in a chemical fume hood. The samples were mounted on stubs and coated with gold before being analyzed with the SEM.

A Zetasizer (Nano ZS90, Malvern Panalytical Malvern, UK) was used to measure the point of zero charge (pH_pzc_) of the C_nc_ and bacteria. The cell solution was prepared by mixing half of the colony in 5 mL of 10 mM NaCl solution [40]. The surface areas of the C_nc_ and IC_nc_ were examined using a Multipoint surface area analyzer (BET) (TriStar II 3020, Micromeritics Inc., Norcross, GA, USA).

### 2.5. Adsorption and Paraquat Biodegradation Using Ceramic, Nanoceramic and Bio−Nanoceramic

An amount of 30 g of C_c_ (without cells), C_nc_ (without cells), or IC_nc_ was experimented on in a batch reactor, a 1500 mL beaker (Pyrex^®^) with an LED digital magnetic hotplate stirrer (TOPTION Instruments, China) under 28 °C for 24 h. The 1 L of investigated synthetic medium consisted of 0.057 g NH_4_PO_4_, 0.017 g K_2_HPO_4_, 0.043 g NaCl, and 0.043 g MgSO_4_. 7H_2_O in distilled water with 10 mg L^−1^ paraquat (5.6 mg L^−1^ as C, 1.1 mg L^−1^ as N). The liquid samples were withdrawn periodically during the 10 h experimental period. The samples were put through a 0.22 µm pore−size membrane filter (MF−Millipore™), and the filtrates were used for the residual paraquat concentration analysis. For dissolved inorganic nitrogen (DIN) species analysis, i.e., ammonium nitrogen, nitrite nitrogen, nitrate nitrogen, and total dissolved nitrogen, the solution samples were filtered using a 0.45 µm pore−size nylon syringe (EZFlow^®^). All experiments were triplicated, and the minimum and maximum values were reported. In addition, the amount of immobilized cells on IC_nc_ and leaching cells in the synthetic solution at the initial and final treatment time (24 h) of the experiment were determined by the plate count technique [41].

(3) $$\%\;cell\;leaching=\frac{final\;amount\;of\;free\;cells\;in\;solution\times 100}{initial\;amount\;of\;immobilized\;cells\;on\;{IC}_{nanoceramic}}$$

Paraquat biodegradation kinetics, which included zero−order, first−order, and second−order kinetics (Equations (4)–(6)), were used for the kinetic parameter calculations [42].

(4) $$\mathrm{Zero}-\mathrm{order}\;\mathrm{kinetics}:\;\left[C\right]=-kt+[C_{0}]$$

(5) $$\mathrm{First}-\mathrm{order}\;\mathrm{kinetics}:\;\ln\left[\frac{C}{C_{0}}\right]=-kt$$

(6) $$\mathrm{Second}-\mathrm{order}\;\mathrm{kinetics}:\;1/[C]=kt+1/[C_{0}]$$

where *C*_0_ is the initial concentration, *C* is the concentration at time *t*, and *k* is the biodegradation rate constant.

### 2.6. DON Biodegradation Degree 

The degradation experiment was conducted in the same manner as mentioned in Section 2.5. Free cells, IC_nc_−P, and IC_nc_−B were performed. Liquid samples were withdrawn periodically 30 mL/time for dissolved inorganic nitrogen (DIN) species analysis, i.e., ammonium nitrogen, nitrite nitrogen, nitrate nitrogen, and total dissolved nitrogen. The solution samples were separated through a 0.45 µm pore−size nylon syringe filter (EZFlow^®,^ VWR, Atlanta, GA, USA). All experiments were in triplicate, and both the minimum and maximum values are presented.

### 2.7. Analytical Methods

For the analysis of paraquat concentration, liquid chromatography−mass spectrometry, (LC−MS) (Agilent 6120, Santa Clara, CA, USA) was performed. Poroshell 120 HILIC−Z (Santa Clara, CA, USA), 2.1 × 100 mm, 2.7 µm (Agilent No.685775−924) was used for the chromatography column. The paraquat analysis was conducted following the USEPA 549.2 method from the U.S. Environmental Protection Agency for the analysis of paraquat and diquat with reversed phase/ion−pair extraction C8 SPE cartridges followed by ion−pair liquid chromatography. The paraquat retention time was 15 min and the detection limit of the paraquat was 0.01 mg L^−1^. 

Focusing on the concentrations of dissolved inorganic nitrogen (DIN) species, the cadmium reduction method was performed to measure NO_3_−N [43], and NO_2_−N and NH_3_−N were measured by the colorimetric and the phenate methods, respectively [44,45]. For TDN analysis, the persulfate chemical wet oxidation method was used [45]. dissolved organic nitrogen (DON) was calculated as the difference between measured total dissolved nitrogen (TDN) and the sum of measured DIN species using Equation (7) [41]. The DON biodegradation degree through the cell−immobilized ceramics was determined as in Equation (8) [41].

*DON* (mg L^−1^ *as N*) = *TDN* − [(*NH*_3_ − *N*) + (*NO*_3_ − *N*) + (*NO*_2_ − *N*)] (7)

*DON_t_ biodegradation degree* (%) = [((*DON_i_* − *DON_t_*) − (*DON_bi_* − *DON_bt_*))/*DON_i_*] × 100% (8)

where *DON_i_* and *DON_t_* are DON before and after the paraquat treatment at time *t*, respectively. *DON_bi_* and D*ON_bt_* are DON before and after paraquat treatment at time *t* for the control (sterile synthetic medium).

### 2.8. Statistical Analysis

To determine the reliability and significance of the findings, a statistical analysis was performed on the experiment results. All experiments were conducted in triplicate, and the results are presented as the mean standard deviation (SD). We used the coefficient of determination (R^2^) and the residual sum of squares to evaluate the biodegradation kinetics models’ fit (RSS). For the observed data, the model with the highest R^2^ and the lowest RSS was deemed to be the best fit. The SPSS statistical software package was utilized for statistical analysis. Excel was used to construct graphs.

## 3. Results and Discussion

### 3.1. Characteristics of Ceramic and Cell−Immobilized Ceramic

**Figure 1:**
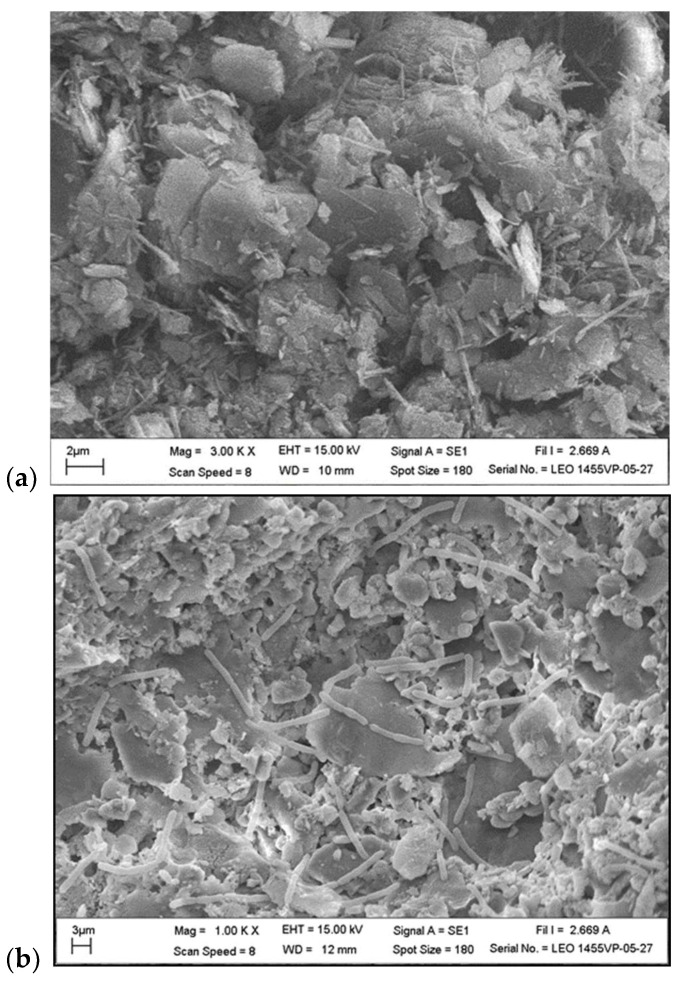

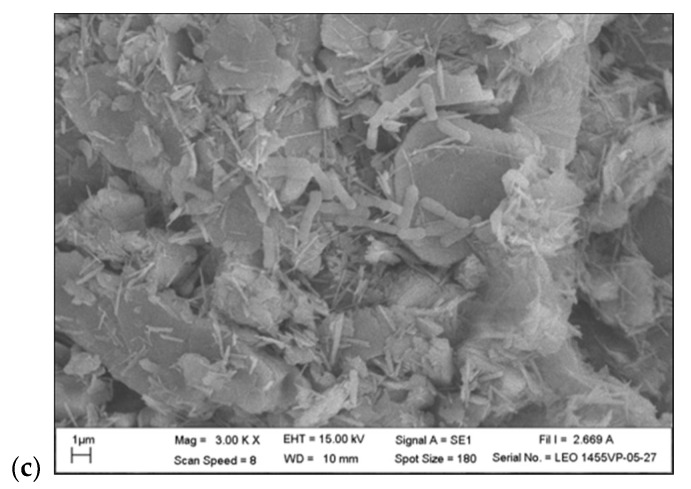
SEM images of (**a**) C_nc_, (**b**) IC_nc_−P, and (**c**) IC_nc_−B.

The SEM images of the surface morphologies for C_nc_ and IC_nc_ are shown in Figure 1. The bacilli or rod shape of both *P. putida* and *B. subtilis* is clearly observed on the surface of the cell−immobilized nanoceramics with *P. putida* (labeled as IC_nc_−P) in Figure 1b, and *B. subtilis* (labeled as IC_nc_−B) in Figure 1c. The rod−shaped bacteria do not appear in C_nc_, as shown in Figure 1a. The surface area of nanoceramics without cells or C_nc_ was 12.95 m^2^/g. In comparison with cell−immobilized nanoceramics, this value is lower due to the bacteria coverage on the ceramic surface. The surface areas of the cell−immobilized nanoceramics were 7.95 and 8.42 m^2^/g for IC_nc_−P and IC_nc_−B, respectively. 

The pH_pzc_ values of C_nc_, *P. putida*, and *B. subtilis* were 1.5, 2.4, and 0.4, respectively (Figure S1a,b). With the immobilization of *P. putida* and *B. subtilis* on the nanoceramic surface, the pH_pzc_ values of IC_nc_−P and IC_nc_−B are slightly changed to 2.6 and 1.0, respectively (Figure S1c).

Apparently, at neutral pH (pH 7), the surface of C_nc_ was negatively charged (negative zeta potential of −21.0 mV). Many negative charged sites are most likely caused by the deprotonation of hydroxyl groups at the edges of the nanoceramic surface, for example −Si−O−Al−O− [46]. This surface charge promoted a strong attachment between the nanoceramic surface and the bacterial cells. The ceramic surface was negatively charged and contained the hydroxyl group at the broken edges of the kaolinite clay [47]. The bacterial surface was dominated by a negative charge and many functional groups, (i.e., hydroxyl, carboxyl, phosphoryl, and amide groups) [48]. Thus, the attachment of immobilized cells on IC_nc_ likely arose from the interactions of the electrostatic force and hydrogen and covalent bonding between the cells and nanoceramics.

### 3.2. Paraquat Adsorption Using Nanoceramics

**Figure 2:**
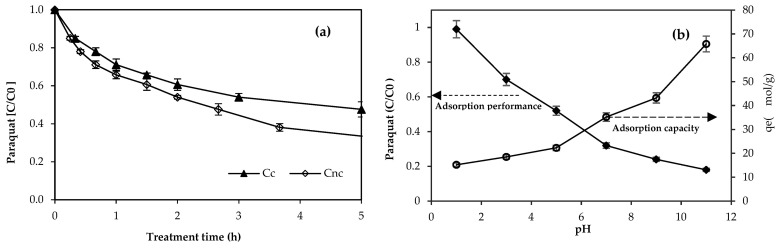
(**a**) Residual paraquat (*C*/*C*_0_) in synthetic medium using nanoceramic (C_nc_) and ceramic (C_c_) via adsorption at pH 7 and (**b**) adsorption capacity (*q_e_*) and residual paraquat (*C*/*C*_0_) in synthetic medium with pH 1–pH 11.

The efficiency of paraquat removal using C_nc_ at pH 7 is shown in Figure 2a. For comparison, the removal performance using ceramic formed by the kaolinite clay (labeled as C_c_) was also investigated. The paraquat in the solution gradually decreased in both cases. Apparently, the C_nc_ provided a higher performance in paraquat adsorption than C_c_. After 5 h, the paraquat removal reached 68% and 52% using C_nc_ and C_c_, respectively. The results clearly indicated that using the nanoclay to produce the ceramic matrices positively influenced paraquat adsorption. Nanoclay made an excellent adsorbent to remove organics and heavy metals [20,21,22,23]. Rezvani and Taghizadeh (2018) [24] demonstrated that the nanoceramic granules provide excellent removal of several water pollutants such as lead, arsenic, and nitrate, and turbidity. The superb ability in paraquat adsorption of C_nc_ (12.95 m^2^/g) in this work is possibly due to a higher specific surface of this material than that of the C_c_ (7.35 m^2^/g). 

In addition, the surface charge of C_nc_ and C_c_ as measured by pH_pzc_ and negative zeta potential was also another important factor influencing the adsorption efficiency of both ceramics. From this work, the pH_pzc_ values of C_nc_ and C_c_ were 1.5 and 2.5, respectively. At experimented pH (pH 7) for paraquat adsorption, the negative zeta potentials of C_nc_ and C_c_ were −21.0 mV and −12.34 mV, respectively. Apparently, the negatively charged surfaces of nanoceramic can promote a stronger binding attachment between the nanoceramic surface and the paraquat than those occurred on the ceramic surface. This surface chemistry possibly resulted from the electrostatic force, and hydrogen and covalent bonding between paraquat and nanoceramics. To enhance the synergistic effect between adsorption and biodegradation, the nanoceramic material was chosen as the microbial support for paraquat degradation and mineralization by cell−immobilized nanoceramics, as discussed in the next section.

**Figure 3:**
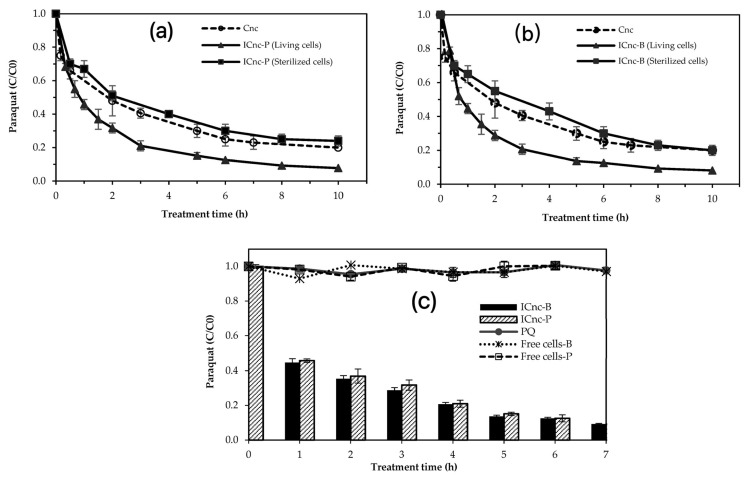
Paraquat removal by C_nc_ (without cells), IC_nc_ (Living cells), and IC_nc_ (Sterilized cells) for (**a**) *P. putida*, (**b**) *B. subtilis*, and (**c**) Paraquat degradation using free cells and cell−immobilized ceramics of *P. putida* and *B. subtilis*.

The solution’s pH effect on the nanoceramics was also investigated. The residual paraquat under the influence of pH using C_nc_ adsorbent is illustrated in Figure 2b. When the solution pH increased from pH 1.0 to pH 11, paraquat removal performance obviously increased and the highest removal of paraquat by C_nc_ occurred at a pH of 11.0. Because the solution pH was much more than the pH_pzc_ (at 1.5), the negatively charged sites of the nanoceramic surface can lead to the electrostatic attraction with the positively charged sites of paraquat molecules. The small fraction of paraquat removal with the acidic solution pH could be ascribed by the electrostatic repulsion between the surface charge of the adsorbent and the cation of paraquat surface. The adsorption capacity (q_e_) of paraquat from each pH is also included in Figure 3b. Apparently, the paraquat adsorption capacity decreased as the solution pH increased. At a high pH (in basic region), the increasing amount of negatively charged sites of the nanoceramic caused an increase in the adsorbed paraquat. This behavior was in good agreement with the previous work using modified bentonite clay for paraquat removal from an aqueous solution [49]. The paraquat adsorption onto the clay surface was an exothermic and a spontaneous process. The maximum value of the equilibrium amount of adsorbed paraquat on the nanoceramic at pH 11 at 65.8 μmol/g was slightly lower than the illite (72.3 μmol/g) and clay mineral (73.1 μmol/g) at the same pH [19,50].

### 3.3. Role of Nanoceramics and Immobilized Cells on Paraquat Degradation

Figure 3 shows paraquat removal in the synthetic medium by the IC_nc_. The paraquat removal readily occurred by both IC_nc_−P and IC_nc_−B, while the Cn_anoceramic_ (without cells) could remove paraquat gradually. The 10 mg L^−^^1^ initial concentration of paraquat was decreased to approximately 1 mg L^−^^1^ within 6 h by both IC_nc_−P and IC_nc_−B. In contrast, after 6 h, the initial concentration of paraquat remaining in the synthetic medium treated by C_nc_ was approximately 28%. Cell immobilization on nanoceramics enhanced paraquat removal, compared to nanoceramics without immobilized cells (C_nc_).

The paraquat removal efficiency of C_nc_, IC_nc_−P, and IC_nc_−B reached 30%, 29%, and 28%, respectively, within 30 min. Results show that the adsorption plays a more important role than biodegradation at the beginning of the paraquat removal. The adsorption process could explain the instant removal of paraquat by the IC_nc_−P and IC_nc_−B at the early stage of the experiment. The adsorption mechanism could refer to the attraction and repulsion of the paraquat molecule and the surface charge of the nanoceramics during the experiment. Recalling point of zero charge values of C_nc_, IC_nc_−P, and IC_nc_−B were 1.5, 2.6, and 1.0, respectively; negatively charged surfaces of nanoceramics are observed when the solution pH is higher than pH_pzc_. As the pK_a_ of cationic paraquat was approximately 9–9.5 cationic [51], the positive charge of the molecule is prominent at neutral solution (pH 7). Consequently, the positive paraquat molecule promptly adsorbed on the negatively charged surface of the ceramic. Chen et al. (2013) [52] conducted cell immobilized on the calcium alginate beads impregnated with activated carbon fiber and concluded that the adsorption on the solid surface is associated with the removal of pollutant during the beginning stage, and this is in good agreement with our results in this study. Nanoceramics removed approximately 72% of paraquat within 10 h. The result echoes paraquat adsorption onto a ceramic surface through electrostatic interaction. The efficiency of clay minerals, such as kaolinite, zeolite, and montmorillonite, as an absorbent in removing paraquat from an aqueous solution has been reported in many studies [15,16,17,18,19].

The synergistic effect between the adsorption and biodegradation was clearly observed after 30 min (Figure 3). The paraquat concentrations in the reactors with IC_nc_–P and IC_nc_−B were more rapidly reduced than that with C_nc_. Interestingly, the immobilized cells on the nanoceramics could subsequently degrade the absorbed paraquat. A similar occurrence was observed in the removal of other pollutants by Lin et al. (2010) [53] and Massalha et al. (2010) [54]. Lin et al. (2010) [53] reported that the immobilization technique significantly enhanced the efficiency of pyridine degradation by Paracoccus sp. strain KT−5. The mixture of clay and AC contributes to the cell immobilization and enhances phenol at high concentrations, as shown by Massalha et al. (2010) [54].

To clearly illustrate the role of the microbial cells and nanoceramics in this process, the sterilized cells of *P. putida* (IC_nc_−P) and *B. subtilis* (IC_nc_−B) were applied for paraquat removal, as shown in Figure 3a and Figure 3b, respectively. Among the three materials consisting of C_nc_, IC_nc_ (Living cells), and IC_nc_ (Sterilized cells), the sterilized cells provided the lowest efficiency in paraquat removal. With the dead cells occupying the nanoceramic surface, paraquat removal efficiency was less than that of the pristine nanoceramics. In addition, the paraquat adsorption ability of the nanoceramic with sterilized cells was also lower than the control nanoceramic (without cells). This result supports the significance of the synergistic effect between the physical adsorption from the nanoceramics and the biodegradation from the cell−immobilized nanoceramics. 

The biodegradation performances of paraquat by the free cells of *P. putida* and *B. subtilis* in comparison with the immobilized cells on nanoceramics of the same microbial species are illustrated in Figure 3c. No biodegradation of free cells was clearly seen. Evidently, the solid phase (e.g., the C_nc_), microbial phase (e.g., the immobilized bacteria), and aqueous phase (i.e., water or paraquat solution) influenced the degradation mechanism of ceramic–bacteria–paraquat interaction. The nanoceramic was a superb support material, providing a habitat for the immobilized microbial cells, in addition to being a superior adsorbent for paraquat through the electrostatic force between the positive charge and negative charge of paraquat and nanoceramic surface, respectively. Thus, the paraquat−degrading bacteria could release the extracellular enzymes to degrade the adsorbed paraquat into smaller molecule(s). Kopytko et al. (2002) [33] also described paraquat degradation by *P. putida* with an activated carbon (AC) support material and the addition of nutrient broth. During 72 h, more than 95% and 47% of the paraquat was removed with and without AC support, respectively. The results of this study are in line with the previous work by Li et al. (2022) [55]. Li et al. (2022) [55] discovered that immobilized Stenotrophomonas acidaminiphila Y4B cells degraded glyphosate more effectively than their free counterparts. Initially, between 0 and 3 days, the glyphosate degradation rate of free cells was faster than that of immobilized cells; however, after 3 days, immobilized cells degraded glyphosate faster than free cells. Immobilized cells were unable to directly touch glyphosate at the beginning of the breakdown process, resulting in a delay. However, the immobilized cells were ultimately more effective than the loose cells [55].

### 3.4. Influence of Paraquat Toxic Stress on Immobilized Cells on Nanoceramic Surface

Toxic stress on the immobilized cells were obtained from the cell leaching test. The initial numbers of immobilized cells on the nanoceramics and free cells in the aqueous solution during the paraquat biodegradation experiments using IC_nc_−P are shown in Table 1. The leaching of initial immobilized cells in the aqueous solution was evaluated 24 h after the paraquat biodegradation experiment. Results showed that the initial amount of immobilized cells on IC_nc_−P was 9.7 × 10^6^ CFU mL^−^^1^, and the residual amounts remaining on the nanoceramics and the free cells in the paraquat aqueous solution after 24 h were 2.0 × 10^6^ and 4.1 × 10^5^ CFU mL^−^^1^, respectively. The cell leaching from the IC_nc_−P in the aqueous solution (without paraquat) is also measured and compared in the same table. The percentages of cell leaching from the IC_nc_−P were 4.23 ± 0.78% and 2.37 ± 0.98% for with and without paraquat in aqueous solution, respectively. 

The paraquat toxic stress demonstrated in this work tentatively occurred from the interaction between reducing agents such as ascorbic acid or oxidoreductase enzymes and the paraquat (PQ^2+^). The paraquat−free radicals (PQ^+•^) generated from the reduction of PQ^2+^ can react with O_2_ to form superoxide anion radicals (O_2_^•−^), which can further be transformed to hydroxyl radicals (OH^•^), as reported previously by Du (2005) [1]. These generated radicals (O_2_^•−^ and OH^•^) induced the oxidative stress to bacteria and caused microbial stress to the system. The toxic stress to the viable microbial cells (for both free cells and immobilized cells) from paraquat at a concentration of 10 mg L^−^^1^ was shown in this work. The presence of paraquat in the solution is greatly influenced by the biofilm structure and the cellular stress response. The leaching percentage of *P. putida* cells from nanoceramics was more pronounced from the effect of paraquat toxic stress. After 24 h of the paraquat biodegradation process, the leaching of initial immobilized cells of *B. subtilis* (3.06 ± 1.12%) in the aqueous solution was also detected. 

Both the *P. putida* and the *B. subtilis* strains are renowned for their metabolic capabilities and environmental adaptability as well as their ability to degrade a wide variety of organic contaminants [32]. From Figure 3, the free cells of both strains restrict the biodegradation performance of paraquat. For the immobilized cells, the paraquat toxic stress affected the leaching of immobilized cells to the aqueous solution; however, the efficiency in paraquat biodegradation remained high. The superior biodegradation of paraquat using immobilized cells, derived from the best adsorbent characteristics of the nanoceramic surfaces, can minimize the direct interaction between paraquat and microbial cells. In addition, the immobilized cell can provide continuous biodegradation, thereby overcoming the disadvantage of limited adsorption capacity and enhancing the survival resilience of bacteria protected from the application environment [30]. Based on the removal of herbicide by immobilized cells by Li et al. (2022) [55], the immobilized cells are more effective in degrading the herbicide due to the fact that they are more protected from the complexity of the natural environment, which can inhibit their activity. Temperature, pH, and the existence of indigenous microorganisms were among these environmental parameters. Consequently, the immobile cells are less susceptible to being washed away by water and attacked by predators.

The paraquat biodegradation using immobilized cells on nanoceramics from this work is also in agreement with the phenol biodegradation using immobilized cells on alginate, clay, and powdered activated carbon from a previous work by Massalha et al. (2010) [54]. The tolerance of the immobilized microbial cells on these support materials allows for excellent mineralization at a phenol concentration that was 2000 mg L^−^^1^ higher than the maximum concentration mineralizable by the free cells. The biofilm adherence to the nanoceramic surface can provide high metabolic activity and strongly resist toxic compounds such as paraquat [31]. 

### 3.5. Kinetics for Paraquat Degradation Using Cell−Immobilized Nanoceramics

Three zero−, first−, and second−order kinetic models determined the trend of paraquat degradation. As shown in Table 2, the second−order model potentially explained the degradation kinetics of paraquat by both IC_nc_−P and IC_nc_−B with R^2^ > 0.95. In addition, the second−order model very well fitted the paraquat removal using C_nc_ with R^2^ > 0.98. A reduction in the half−life of paraquat from 1.3 h to 0.5 h was observed when cell−immobilized nanoceramics were applied for paraquat removal in comparison with the nanoceramics. The initial degradation rate (r) of paraquat using C_nc_−B (0.0092 mg L^−^^1^ min^−^^1^) was slightly higher than the C_nc_−P (0.0090 mg L^−^^1^ min^−^^1^). Similarly, the degradation rate constant (k) of paraquat with C_nc_−B (0.2408 M^−^^1^ min^−^^1^) was marginally greater than the C_nc_−P (0.2126 M^−^^1^ min^−^^1^). The results show that both IC_nc_−P and IC_nc_−B exhibited a higher performance in paraquat removal in comparison with the same biodegradation kinetic models.

### 3.6. Determination of DON Biodegradation Degree Using Cell−Immobilized Nanoceramics

**Figure 4:**
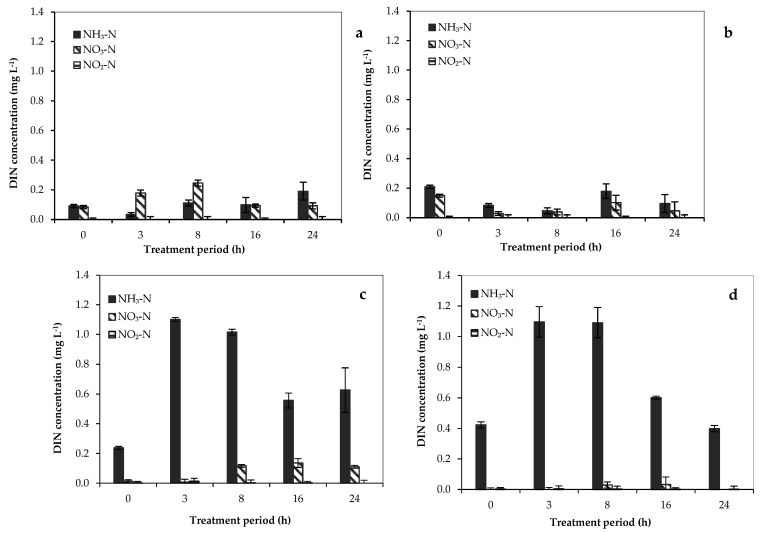
Concentrations of dissolved inorganic nitrogen (DIN) during the biodegradation of paraquat using (**a**) *P. putida* (free cell), (**b**) *B. subtilis* (free cell), (**c**) IC_nc_−P, and (**d**) IC_nc_−B.

The DIN concentrations (NO_3_–N, NO_2_–N, and NH_3_–N) during paraquat biodegradation using *P. putida* (free cells), *B. subtilis* (Free cells), IC_nc_−P, and IC_nc_−B are demonstrated in Figure 4. 

Apparently, the DIN concentrations obtained from paraquat biodegradation between the free cells and immobilized cells of *P. putida* are substantially different. The relatively low performances in the transformation of paraquat to each inorganic nitrogen by the free cells of *P. putida* are presented in Figure 4a,b for *B. subtilis*. Using cell−immobilized nanoceramics (IC_nc_−P and IC_nc_−B), the predominant species of inorganic nitrogen from paraquat biodegradation detected in the system was NH_3_−N. The appearance of NO_3_−N was detected after 8 h of the treatment, while the NH_3_−N concentration continuously decreased. The nitrification occurred in the system because both microbial cells (*P. putida* and *B. subtilis*) are heterotrophic nitrifying bacteria [28,56]. Daum et al. (1998) [56] and Wang et al. (2019) [28] previously demonstrated the ability of heterotrophic nitrifying bacteria (*P. putida*) in oxidizing ammonia to nitrite, then into nitrate, thus these results agree with this study.

**Figure 5:**
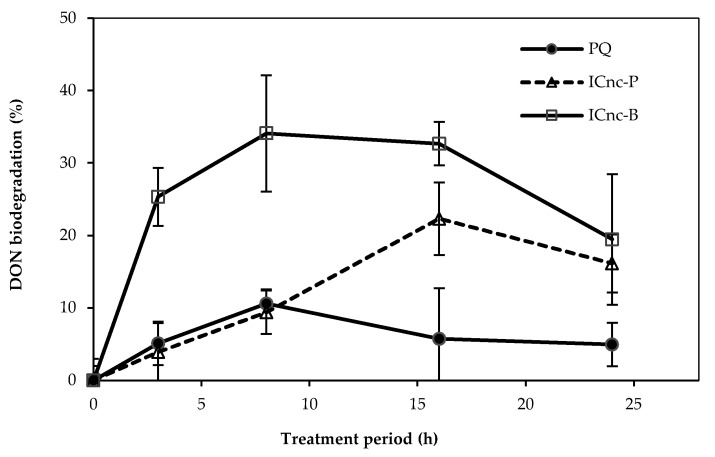
DON biodegradation degree of paraquat using IC_nc_−P and IC_nc_−B.

The DON biodegradation degrees derived from paraquat degradation using the cell−immobilized nanoceramics for both IC_nc_−P and IC_nc_−B are illustrated in Figure 5. The DON biodegradation degrees using IC_nc_−P and IC_nc_−B were 22 ± 1.0% at 16 h and 34 ± 1.3% at 8 h, respectively. The IC_nc_−B provided a higher DON biodegradation degree than the IC_nc_−P. These immobilized cells were able to convert the organic nitrogen in the paraquat molecule to inorganic nitrogen (NH_3_−N and NO_3_−N) through the ammonification. The detection of inorganic nitrogen, especially ammonia, in this work is in good agreement with Dinis−Oliveira et al. (2008) [57]. The intermediate products from paraquat degradation were monoquat and 4−carboxy−1−methylpyridinium (MINA), which were degraded further to smaller molecules, including methylamine, formate, and oxalate, before carbon dioxide, ammonia, and water were obtained as the ultimate products [57]. Regarding to the DON biodegradation degree and the detected inorganic nitrogen species during the paraquat removal, both cell−immobilized nanoceramics (IC_nc_−P and IC_nc_−B) were able to ammonify paraquat, oxidize the ammonia generated, and mineralize the paraquat.

## 4. Conclusions

This study showed the synergy of nanoceramic surface paraquat adsorption and immobilized cell biodegradation and mineralization. Two strains of *P. putida* (IC_nc_−P) and *B. subtilis* (IC_nc_−B) immobilized on nanoceramics removed paraquat better than their free cells. The second−order model accurately describes paraquat degradation kinetics for IC_nc_−P and IC_nc_−B. IC_nc_−B degraded paraquat at 0.0092 mg L^−1^ min^−1^, slightly faster than IC_nc_−P (0.0090 mg L^−1^ min^−1^). Paraquat with IC_nc_−B (0.2408 M^−1^ min^−1^) had a slightly higher degradation rate constant (k) than IC_nc_−P (0.2126 M^−1^ min^−1^). Both heterotrophic nitrifying bacteria possibly convert paraquat into inorganic nitrogen species, including NH_3_−N, NO_3_−N, and NO_2_−N. Approximately 20–35% of organic nitrogen in paraquat was biologically ammonified, demonstrating the strength of the cell−immobilized nanoceramics. For further research, the application of these cell−immobilized nanoceramics is recommended for pesticide removal in wastewater. Organic loading, pH, and dissolved oxygen concentration should be investigated to achieve the high capability of paraquat degradation.

## Figures and Tables

**Table 1:** Amount of cells on ceramics (CFU g^−1^) and in aqueous solution (CFU mL^−1^) during the paraquat biodegradation experiments.

Initial Stage of Experiment (0 h)		Final Stage of Experiment (24 h)	
Immobilized Cells on Nanoceramics	Free Cells in Aqueous Solution	Immobilized Cells on Nanoceramics	Free Cells in Aqueous Solution
IC_nc_−P in aqueous solution (without paraquat)			
7.6 × 10^6^	0	6.0 × 10^6^	1.8 × 10^5^
IC_nc_−P in paraquat aqueous solution			
9.7 × 10^6^	0	2.0 × 10^6^	4.1 × 10^5^
IC_nc_−B in paraquat aqueous solution			
7.2 × 10^5^	0	6.5 × 10^5^	2.2 × 10^4^
Leaching percentage			
IC_nc_−P in aqueous solution (without paraquat) = 2.37 ± 0.98%			
IC_nc_−P in paraquat aqueous solution = 4.23 ± 0.78%			
IC_nc_−B in paraquat aqueous solution = 3.06 ± 1.12%			

**Table 2:** Kinetic parameters for paraquat degradation by cell−immobilized nanoceramics.

Samples	Zero−Order Model			First−Order Model			Second−Order Model		
	Equation, R^2^	k (M min^−1^)	Half−Life (h)	Equation, R^2^	k (min^−1^)	Half−Life (h)	Equation, R^2^	k (M^−1^ min^−1^)	Half−Life (h)
C_nc_	y = −0.1724x + 0.8387 R^2^ = 0.8018	0.1724	25.1	y = 0.3612x R^2^ = 0.7610	0.3612	1.9	y = 0.0865x + 0.1133 R^2^ = 0.9872	0.0865	1.3
IC_nc_−P	y = −0.2289x + 0.7904 R^2^ = 0.8072	0.2289	19.5	y = 0.5817x R^2^ = 0.8931	0.5817	1.2	y = 0.2126x + 0.0696 R^2^ = 0.9566	0.2126	0.5
IC_nc_−B	y = −0.2535x + 0.8287 R^2^ = 0.7879	0.2535	17.0	y = 0.6009x R^2^ = 0.9051	0.6009	1.2	y = 0.2408x + 0.0575 R^2^ = 0.9606	0.2408	0.5

## Data Availability

Data will be made available on request.

## Supplementary Materials

The following supporting information can be downloaded at: https://www.mdpi.com/article/10.3390/toxics11070638/s1, Figure S1: Zeta potential of (**a**) C_nc_, (**b**) bacteria, and (**c**) IC_nc._

## References

[1] USEPA (2013). Paraquat and Diquat in Recognition and Management of Pesticide Poisonings.

[2] Leadprathom N. (2015). Toxicity of Paraquat to Aquatic Animal. J. King Mongkut’s Agric.

[3] Roede J.R., Miller G.W. (2014). Paraquat in Encyclopedia of Toxicology.

[4] Sannino F., Gianfreda L. (2001). Pesticide influence on soil enzymatic activities. Chemosphere.

[5] Sebiomo A., Ogundero V., Bankole S. (2010). Effect of four herbicides on microbial population, soil organic matter and dehydrogenase activity. Afr. J. Biotechnol..

[6] Smith S.N., Pugh G.J.F. (1979). Evaluation of dehydrogenase as a suitable indicator of soil microflora activity. Enzym. Microb. Technol..

[7] Pateiro−Moure M., Pérez−Novo C., Arias−Estévez M., Rial−Otero R., Simal−Gándara J. (2009). Effect of organic matter and iron oxides on quaternary herbicide sorption–desorption in vineyard−devoted soils. J. Colloid Interface Sci..

[8] Rashidipour M., Maleki A., Kordi S., Birjandi M., Pajouhi N., Mohammadi E., Heydari R., Rezaee R., Rasoulian B., Davari B. (2019). Pectin/Chitosan/Tripolyphosphate Nanoparticles: Efficient Carriers for Reducing Soil Sorption, Cytotoxicity, and Mutagenicity of Paraquat and Enhancing Its Herbicide Activity. J. Agric. Food Chem.

[9] Karunarathne A., Gunnell D., Konradsen F., Eddleston M. (2019). How many premature deaths from pesticide suicide have occurred since the agricultural Green Revolution?. Clin. Toxicol..

[10] Blinová L., Sirotiak M. (2021). Utilization of Waste-Based Sorbents for Removal of Pharmaceuticals from Water: A Review. Res. Pap. Fac. Mater. Sci. Technol. Slovak Univ. Technol..

[11] Phuinthiang P., Kajitvichyanukul P. (2018). Degradation of paraquat from contaminated water using green TiO_2_ nanoparticles synthesized from *Coffea arabica* L. in photocatalytic process. Water Sci. Technol.

[12] Sharma I. (2020). Bioremediation Techniques for Polluted Environment: Concept, Advantages, Limitations, and Prospects. Trace Metals in the Environment-New Approaches and Recent Advances.

[13] Ha N.T.H., Toan N.C., Kajitvichyanukul P. (2022). Enhanced paraquat removal from contaminated water using cell-immobilized biochar. Clean Technol. Environ. Policy.

[14] Sarkar B., Xi Y., Megharaj M., Krishnamurti G.S.R., Bowman M., Rose H., Naidu R. (2012). Bioreactive Organoclay: A New Technology for Environmental Remediation. Crit. Rev. Environ. Sci. Technol..

[15] Cheng Y., Lin H.Y., Chen Z., Megharaj M., Naidu R. (2012). Biodegradation of crystal violet using *Burkholderia vietnamiensis* C09V immobilized on PVA–sodium alginate–kaolin gel beads. Ecotoxicol. Environ. Saf..

[16] Martins D., Simões M., Melo L. (2015). Adsorption of paraquat dichloride to kaolin particles and to mixtures of kaolin and hematite particles in aqueous suspensions. J. Water Secur..

[17] Sirival R., Patdhanagul N., Preecharram S., Photharin S. (2018). Removal of paraquat solution onto zeolite material. AIP Conf. Proc..

[18] Tsai W.T., Chen H.R. (2013). Adsorption kinetics of herbicide paraquat in aqueous solution onto a low-cost adsorbent, swine-manure-derived biochar. Int. J. Environ. Sci. Technol..

[19] Tsai W.T., Lai C.W. (2006). Adsorption of herbicide paraquat by clay mineral regenerated from spent bleaching earth. J. Hazard. Mater..

[20] Diallo M., Christie S., Swaminathan P., Johnson J., Goddard W. (2005). Dendrimer Enhanced Ultrafiltration. 1. Recovery of Cu(II) from Aqueous Solutions Using PAMAM Dendrimers with Ethylene Diamine Core and Terminal NH_2_ Groups. Environ. Sci. Technol..

[21] Kahraman H.T., Pehlivan E. (2017). Use of Organo−Montmorillonite Nanoclay as an Environmentally Friendly Adsorbent for Removal of Hexavalent Chromium. Bio. Environ. Eng..

[22] Pandey S. (2017). A comprehensive review on recent developments in bentonite−based materials used as adsorbents for wastewater treatment. J. Mol. Liq..

[23] Prachi, Gautam P., Madathil D., Nair A.N.B. (2013). Nanotechnology in waste water treatment: A review. Int. J. Chem. Tech. Res..

[24] Rezvani P., Taghizadeh M.M. (2018). On using clay and nanoclay ceramic granules in reducing lead, arsenic, nitrate, and turbidity from water. Appl. Water Sci..

[25] Taha M.R., Mobasser S. (2015). Adsorption of DDT and PCB by Nanomaterials from Residual Soil. PLoS ONE.

[26] Yue R., An C., Ye Z., Bi H., Chen Z., Liu X., Zhang X., Lee K. (2022). Cleanup of oiled shorelines using a dual responsive nanoclay/sodium alginate surface washing agent. Environ. Res..

[27] Siripattanakul S., Wirojanagud W., McEvoy J., Khan E. (2008). Effect of Cell-to-matrix Ratio in Polyvinyl Alcohol Immobilized Pure and Mixed Cultures on Atrazine Degradation. Water Air Soil Pollut. Focus.

[28] Wang X.H., Lei Y., Ren Y.X., Chen N., Xiao Q., Cui S., Li D. (2019). Nitrogen Removal by Heterotrophic Nitrifying Bacterium *Pseudomonas putida* YH and Its Kinetic Characteristics. Huan Jing Ke Xue Huanjing Kexue.

[29] Lou L., Huang Q., Lou Y., Lu J., Hu B., Lin Q. (2019). Adsorption and degradation in the removal of nonylphenol from water by cells immobilized on biochar. Chemosphere.

[30] Wu C., Zhi D., Yao B., Zhou Y., Yang Y., Zhou Y. (2022). Immobilization of microbes on biochar for water and soil remediation: A review. Environ. Res..

[31] Cassidy M.B., Lee H., Trevors J.T. (1996). Environmental applications of immobilized microbial cells: A review. J. Ind. Microbiol..

[32] Huang Y., Zhan H., Bhatt P., Chen S. (2019). Paraquat Degradation From Contaminated Environments: Current Achievements and Perspectives. Front. Microbiol..

[33] Kopytko M., Chalela G., Zauscher F. (2002). Biodegradation of two commercial herbicides (Gramoxone and Matancha) by the bacteria *Pseudomonas putida*. Electron. J. Biotechnol..

[34] Tu C., Bollen W. (2006). Interaction between paraquat and microbes in soils. Weed Res..

[35] Wu C., Liu J., Chen S., Deng X., Li Q. (2013). Isolation and Characterization of Paraquat-Degrading Extracellular Humus-Reducing Bacteria from Vegetable Field. Adv. Mater. Res..

[36] Viriyawattana N., Sinworn S. (2014). Biodegradation of paraquat by the novel bacterial strain *aeromonas Veronii* NK67 from cassava fields in Thailand. Asian J. Microbiol. Biotechnol. Environ. Sci..

[37] Teerakun M. (2020). Optimization of paraquat degradation microbial consortium from contaminated soil using statistic method. GEOMATE J..

[38] Harley J.P., Lansing M.P. (2004). Laboratory Exercises in Microbiology.

[39] Kang C., Wu P., Li L., Yu L., Ruan B., Gong B., Zhu N. (2017). Cr(VI) reduction and Cr(III) immobilization by resting cells of *Pseudomonas aeruginosa* CCTCC AB93066: Spectroscopic, microscopic, and mass balance analysis. Environ. Sci. Pollut. Res..

[40] Rochex A., Lecouturier D., Pezron I., Lebeault J.M. (2004). Adhesion of a *Pseudomonas putida* strain isolated from a paper machine to cellulose fibres. Appl. Microbiol. Biotechnol..

[41] Jindakaraked M., Khan E., Kajitvichyanukul P. (2021). Biodegradation of paraquat by *Pseudomonas putida* and *Bacillus subtilis* immobilized on ceramic with supplemented wastewater sludge. Environ. Pollut..

[42] Connors K.A. (1990). Chemical Kinetics: The Study of Reaction Rates in Solution.

[43] Jones M.N. (1984). Nitrate reduction by shaking with cadmium: Alternative to cadmium columns. Water Res..

[44] APHA (2005). Standard Methods for the Examination of Water and Wastewater.

[45] Bronk D.A., Lomas M.W., Glibert P.M., Schukert K.J., Sanderson M.P. (2000). Total dissolved nitrogen analysis: Comparisons between the persulfate, UV and high temperature oxidation methods. Mar. Chem..

[46] de Fuentes I.E., Viseras C.A., Ubiali D., Terreni M., Alcántara A.R. (2001). Different phyllosilicates as supports for lipase immobilisation. J. Mol. Catal. B Enzym..

[47] An N., Zhou C.H., Zhuang X.Y., Tong D.S., Yu W.H. (2015). Immobilization of enzymes on clay minerals for biocatalysts and biosensors. Appl. Clay Sci..

[48] Tourney J., Ngwenya B.T. (2014). The role of bacterial extracellular polymeric substances in geomicrobiology. Chem. Geol..

[49] Rasaie A., Sabzehmeidani M.M., Ghaedi M., Ghane−Jahromi M., Sedaratian−Jahromi A. (2021). Removal of herbicide paraquat from aqueous solutions by bentonite modified with mesoporous silica. Mater. Chem. Phys..

[50] Draoui K., Denoyel R., Chgoura M., Rouquerol J. (1999). Adsorption of Paraquat on minerals: A thermodynamic study. J. Therm. Anal. Calorim..

[51] Lu X., Rasco B., Jabal J., Aston E., Lin M., Konkel M. (2011). Investigating Antibacterial Effects of Garlic (Allium sativum) Concentrate and Garlic-Derived Organosulfur Compounds on Campylobacter jejuni by Using Fourier Transform Infrared Spectroscopy, Raman Spectroscopy, and Electron Microscopy. Appl. Environ. Microbiol..

[52] Chen D.Z., Fang J.Y., Shao Q., Ye J.-X., Ouyang D.J., Chen J.M. (2013). Biodegradation of tetrahydrofuran by *Pseudomonas oleovorans* DT4 immobilized in calcium alginate beads impregnated with activated carbon fiber: Mass transfer effect and continuous treatment. Bioresour. Technol..

[53] Lin Q., Donghui W., Jianlong W. (2010). Biodegradation of pyridine by *Paracoccus* sp. KT-5 immobilized on bamboo−based activated carbon. Bioresour. Technol..

[54] Massalha N., Shaviv A., Sabbah I. (2010). Modeling the effect of immobilization of microorganisms on the rate of biodegradation of phenol under inhibitory conditions. Water Res..

[55] Li J., Chen W.J., Zhang W., Zhang Y., Lei Q., Wu S., Huang Y., Mishra S., Bhatt P., Chen S. (2022). Effects of Free or Immobilized Bacterium *Stenotrophomonas acidaminiphila* Y4B on Glyphosate Degradation Performance and Indigenous Microbial Community Structure. J. Agric. Food Chem..

[56] Daum M., Zimmer W., Papen H., Kloos K., Nawrath K., Bothe H. (1998). Physiological and molecular biological characterization of ammonia oxidation of the heterotrophic nitrifier *Pseudomonas putida*. Curr. Microbiol..

[57] Dinis−Oliveira R.J., Duarte J.A., Sánchez−Navarro A., Remião F., Bastos M.L., Carvalho F. (2008). Paraquat poisonings: Mechanisms of lung toxicity, clinical features, and treatment. Crit. Rev. Toxicol..

