# Supplementary material for: Biodegradation Capabilities of Paraquat-Degrading Bacteria Immobilized on Nanoceramics
Source: Toxics. 2023 Jul 23;11(7):638. doi: 10.3390/toxics11070638 (PMC10386355; doi:10.3390/toxics11070638)
Supplement: Supplementary file 1 [file toxics-11-00638-s001.zip › toxics-2479949-supplementary.pdf]

Supplementary Materials

Biodegradation Capabilities of Paraquat–Degrading Bacteria Immobilized on Nanoceramics

Manee Jindakaraked [1], Eakalak Khan [2] and Puangrat Kajitvichyanukul [1,*]

[1] Department of Environmental Engineering, Faculty of Engineering, Chiang Mai University, Chiang Mai 52000, Thailand; rammy55063390@gmail.com
[2] Civil and Environmental Engineering and Construction Department, University of Nevada, Las Vegas, NV 89154-4015, USA; eakalak.khan@unlv.edu
* Correspondence: kpuangrat@gmail.com or puangrat.k@cmu.ac.th

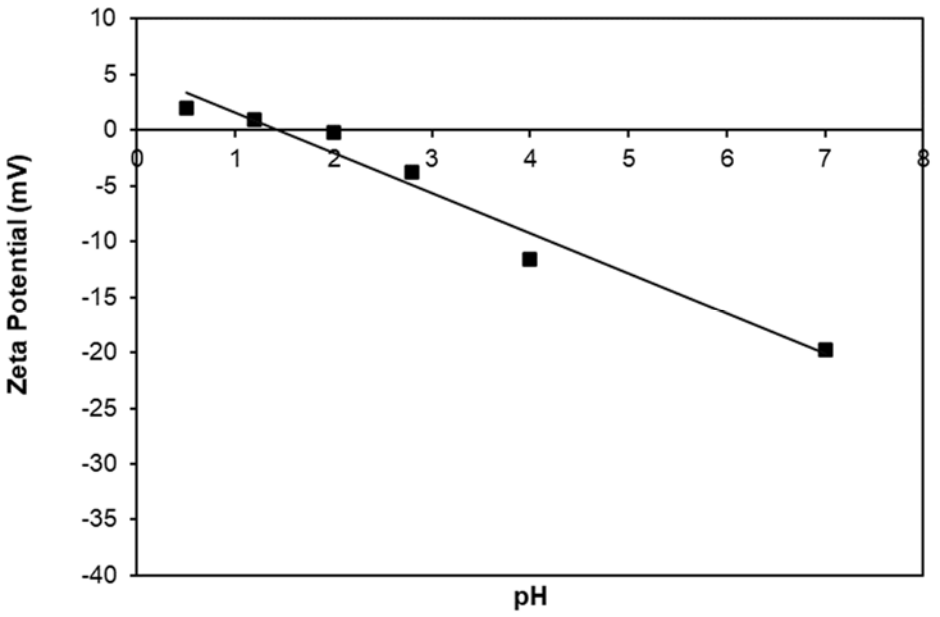
Zeta Potential (mV)
pH
10
5
0
-5
-10
-15
-20
-25
-30
-35
-40
0
1
2
3
4
5
6
7
8

(a)

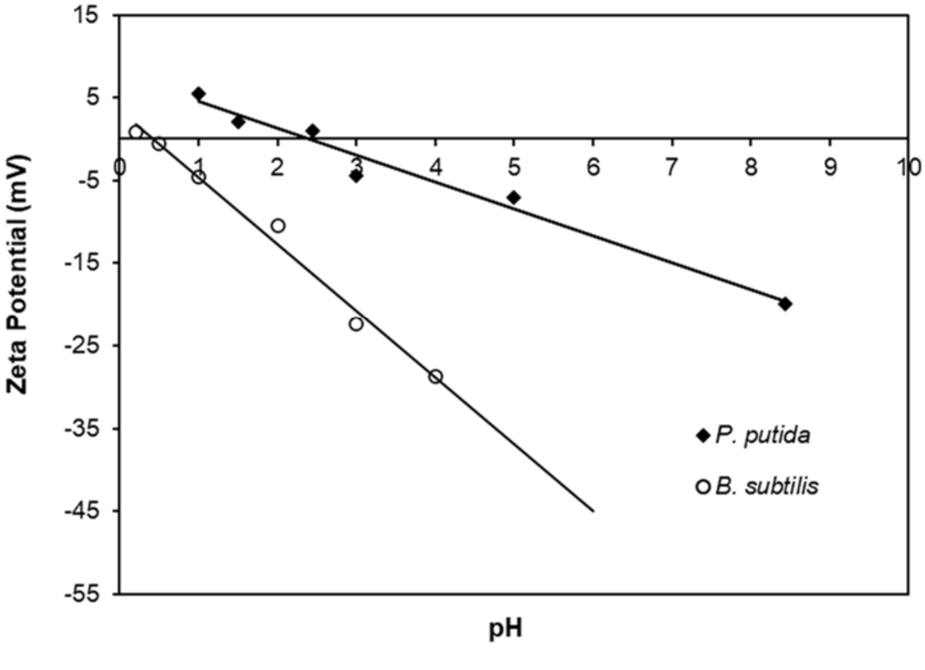
Zeta Potential (mV)
pH
P. putida
B. subtilis
15
5
-5
-15
-25
-35
-45
-55
0
1
2
3
4
5
6
7
8
9
10

(b)

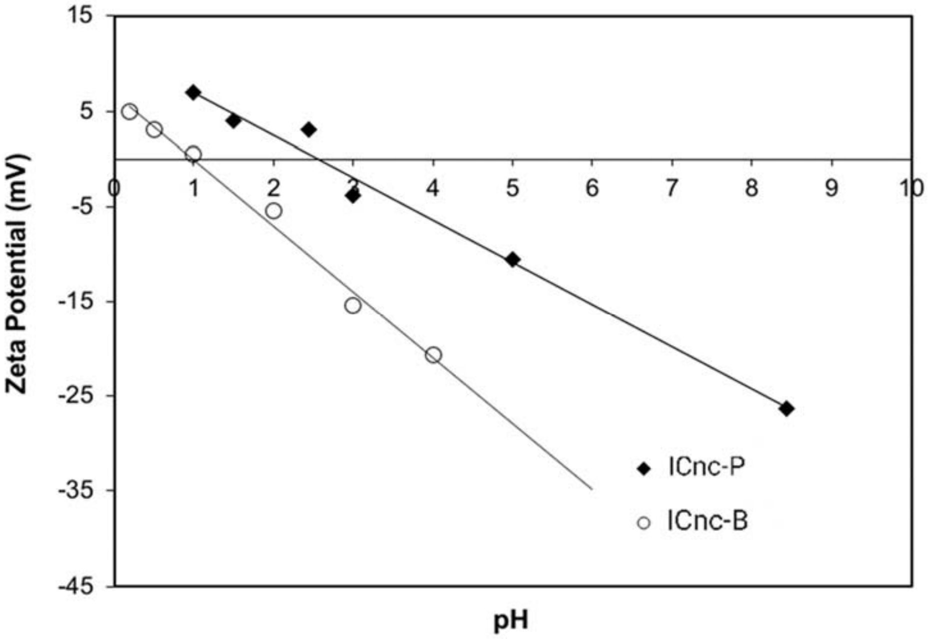

(c)

Figure S1. Zeta potential of (a) C_{nc}, (b)bacteria, and (c) IC_{nc}.
